# Tracking of dietary patterns from early childhood to school age in the Brazilian Food and Nutritional Surveillance System, 2008–2019

**DOI:** 10.1017/S1368980026102663

**Published:** 2026-07-06

**Authors:** Giesy Ribeiro Souza, Andreia Oliveira, Milton Severo, Priscila Ribas de Faria Costa, Dayana Rodrigues Farias, Bárbara H. Lourenco, Lais Silva Sacramento, Leila Denise Alves Ferreira Amorim, Rita de Cássia Ribeiro-Silva, Maurício Lima Barreto

**Affiliations:** 1 Center for Data Integration and Knowledge for Health, Gonçalo Moniz Institute, Oswaldo Cruz Foundation, Salvador, BA, Brazil; 2 Institute of Collective Health, Federal University of Bahia, Salvador, BA, Brazil; 3 EPIUnit - Instituto de Saúde Pública, Universidade do Porto [EPIUnit - Institute of Public Health, University of Porto], Porto, Portugal; 4 Laboratório para a Investigação Integrativa e Translacional em Saúde Populacional (ITR), Universidade do Porto [Laboratory for Integrative and Translational Research in Population Health, University of Porto], Porto, Portugal; 5 Department of Public Health and Forensic Sciences and Medical Education, Faculty of Medicine, University of Porto, Porto, Portugal; 6 School of Nutrition, Federal University of Bahia, Salvador, BA, Brazil; 7 Nutritional Epidemiology Observatory, Josué de Castro Nutrition Institute, Federal University of Rio de Janeiro, Rio de Janeiro, RJ, Brazil; 8 Department of Nutrition, School of Public Health, University of São Paulo, São Paulo, SP, Brazil; 9 Institute of Mathematics and Statistics, Federal University of Bahia, Salvador, BA, Brazil

**Keywords:** Dietary pattern tracking, Ultra-processed food, Longitudinal dietary patterns, Latent transition analysis, Breast-feeding, Nutrition

## Abstract

**Objective:**

(i) Define children’s dietary patterns at three developmental stages – early childhood (1 to < 3 years), preschool age (3 to < 6 years) and school age (6 to < 11 years) – and assess transitions over time and (ii) verify the association between exposure to exclusive breast-feeding and the trajectory of patterns.

**Design:**

Cohort study using 2008–2019 data from the Brazilian Food and Nutritional Surveillance System.

**Setting:**

Standardised markers of previous-day food intake identified dietary patterns. Latent transition analysis assessed shifts over time and estimated the effect of exclusive breast-feeding for 3–6 months on dietary pattern transitions.

**Participants:**

135 340 children.

**Results:**

Two dietary patterns emerged: higher ultra-processed food intake and lower ultra-processed food intake. Patterns showed notable stability over time. Exclusively breastfed children following a lower ultra-processed food pattern in early childhood had 11 % higher odds (OR = 1·11, 95 % CI = 1·06, 1·16) of maintaining this pattern at preschool age. Exclusively breastfed children in the lower ultra-processed food pattern had a 10 % lower likelihood (OR = 0·90, 95 % CI = 0·86, 0·95) of transitioning to a higher ultra-processed food pattern at preschool age.

**Conclusion:**

Dietary patterns in Brazilian children showed significant stability from early childhood to school age. Exclusive breast-feeding may protect against transitioning to high ultra-processed food patterns.

Monitoring children’s dietary intake and other health behaviours is essential to guiding actions focused on promoting and establishing adequate and healthy eating habits and their maintenance in later years^(1,2)^. The need to monitor dietary habits among children has intensified in recent years due to the growing epidemic of overweight and associated diseases among young people worldwide^(3,4)^.

Despite the relevance of maintaining adequate and healthy dietary patterns, studies assessing the tracking of dietary patterns over the lifespan are scarce, making it difficult to evaluate long-term dietary exposure^(5)^. In epidemiology, the term ‘tracking’ refers to the tendency of a variable to remain stable over time^(6)^. In the context of diet, dietary tracking describes the maintenance of nutrient intake or dietary patterns across different stages of life. This concept highlights that eating behaviours acquired in early childhood often persist into adolescence and adulthood^(7)^. Findings from studies with children and adolescents are mixed, with some studies reporting stability^(3,8–10)^, while others show changes in dietary patterns^(11)^. Dietary patterns have been identified in some cross-sectional studies in Brazilian children and adolescents^(1,12)^. None of these studies investigated the stability of these dietary patterns specifically from childhood to school age, nor the factors related to this possible transition.

Thus, this study aimed to achieve the following purposes using data from the Food and Nutritional Surveillance System – SISVAN: (i) to define dietary patterns of children in three developmental windows: early childhood (1 to < 3 years), preschool age (3 to < 6 years) and school age (6 to < 11 years) and to assess transitions over time and (ii) verify the association between exposure to exclusive breast-feeding and the trajectory of patterns from early childhood to school age. These findings should have the potential to assist in developing targeted actions for higher-risk groups and contribute to the prevention and control of diet-related morbidities.

## Methods

### Study design and population

This was a cohort study conducted with data obtained from the Brazilian Food and Nutritional Surveillance System (SISVAN), collected between 2008 and 2019. The SISVAN is part of the national Food and Nutrition Surveillance (FNS) strategies and is responsible for monitoring the nutritional status of the Brazilian population throughout all stages of the life course^(13,14)^. The system compiles information on nutritional status and dietary intake of the population assisted in primary health care within the Unified Health System (SUS), collected routinely according to standardised protocols established by the Ministry of Health^(13)^. These data provide essential support for the formulation of programmes, actions and public policies aimed at promoting adequate and healthy diets, as well as preventing and managing nutrition-related conditions^(15)^.

**Figure 1.**
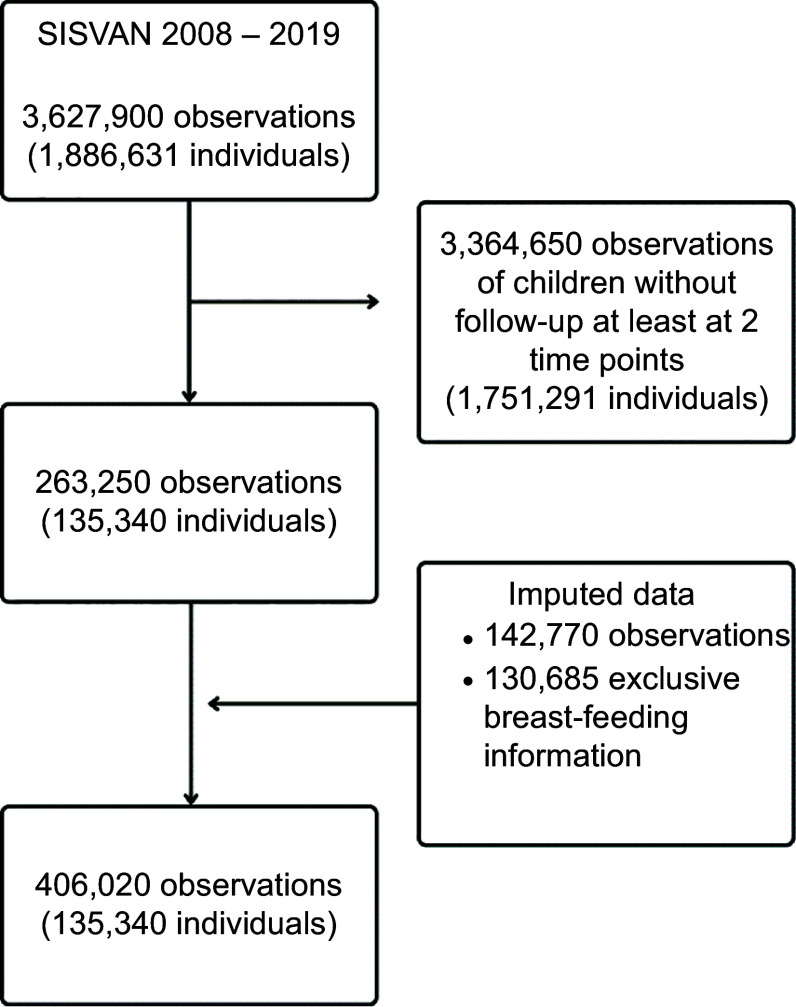
Flow chart of study sample selection – SISVAN, Brazil, 2008–2019.

The study population consisted of children with dietary intake information recorded in SISVAN between 2008 and 2019. Eligibility required the availability of food intake data in at least two distinct life-course stages: early childhood (1 to < 3 years), preschool age (3 to < 5 years) and school age (6 to < 11 years), as well as information on exclusive breast-feeding between 3 and 6 months. For each stage, the most recent observation within a given year was considered. Children without information in at least two stages were excluded (Figure 1).

The cohort was constructed using individualised and anonymised SISVAN data made available by the Ministry of Health to the Center for Data and Knowledge Integration for Health (CIDACS/Fiocruz). Due to the sensitive nature of the information and the need to protect individual privacy, CIDACS cohorts are not publicly accessible. The data used in this study are secondary, anonymised and available for research and policy purposes. Since no personal identifiers were included, informed consent was not required.

CIDACS implements rigorous technical and physical measures for data management, documentation, storage and security, in compliance with the Brazilian General Data Protection Law (Law No. 13.709/2018) and the guidelines of Resolution No. 466/2012 of the National Health Council’s National Research Ethics Commission. This study was approved by the Institutional Review Board of the Collective Health Institute, Federal University of Bahia (UFBA) (CAAE: 41695415.0.0000.5030).

### Study variable

The study population was characterised according to variables available in the SISVAN database: sex (female/male), age group (early childhood, preschool age and school age) and geographic region of residence (North, Northeast, Central-West, Southeast and South). The Brazilian Deprivation Index (BDI) was used as a proxy for the socio-economic profile of each child’s municipality of residence. The BDI is an area-based measure of material deprivation and overall socio-economic position across Brazil’s geographic regions. It is calculated using three indicators: (1) the percentage of households with a per capita income ≤ ½ minimum wage; (2) the percentage of individuals aged ≥ 7 years who are illiterate and (3) the mean percentage of residents with inadequate access to sewage, water or waste collection services, and without a bath or shower. The index is organised into quintiles, ranging from the first quintile (least deprived) to the fifth quintile (most deprived)^(16)^.

### Food consumption

Food consumption was evaluated using a standardised form with specific questions to qualitatively measure food consumption information, used by SUS primary healthcare services. These forms were developed based on the WHO guidelines for assessing infant and young child feeding practices, which were revised and updated in 2015^(17)^. In Brazil, the monitoring of nutritional status is part of the FNS, provided for in the law that created the SUS, which consists of the continuous description of the food and nutrition conditions of the Brazilian population^(13)^. To ensure uniform evaluation across the entire period, we chose to analyse dietary consumption information that remained consistent or equivalent over time, focusing on individuals aged 1 year and older, covering the period from 2008 to 2019. The breast-feeding indicator was directly available in SISVAN until 2014. From 2015 onwards, it was derived based on a ‘yes’ response to the question *‘Did the child drink breast milk yesterday?’* combined with ‘no’ responses to all other questions on the consumption of foods and beverages among children up to 6 months of age. The following information was available: beans; fresh fruits (excluding fruit juice); vegetables and/or greens (excluding potatoes, cassava, yams, taro and sweet potatoes); sweetened beverages (soft drinks, boxed juice, powdered juice, boxed coconut water, guarana/blackcurrant syrups and fruit juice with added sugar); instant noodles, packaged snacks or savoury biscuits; and sandwich cookies, sweets or treats (candies, lollipops, chewing gum, caramels, gelatin and chocolates). The response options available were: ‘yes’, ‘no’ and ‘do not know’ if consumed in the previous day. Responses marked as ‘do not know’ were treated as missing data.

### Statistical analysis

Latent transition analysis (LTA) was used to assess changes in dietary patterns over time. It tracked across latent states, capturing shifts in individuals’ dietary patterns^(18,19)^.

To identify and define the number of classes in children’s dietary patterns, we used latent class analysis (LCA). This is a statistical method used to identify unobserved patterns of behaviour in a data sample from a set of observed variables^(18,20)^. This approach assumes that the studied population consists of distinct subgroups, or latent classes, each with its probabilistic characteristics^(21)^. Individuals are assigned to a class based on their responses to a set of variables^(20)^. The optimal number of classes was determined by comparing Akaike information criterion and Bayesian information criterion, which balance model complexity with data fit^(22)^. Entropy values close to 1 indicated clear differentiation between classes^(18)^. Although 3- and 4-class models had slightly better fit indices, the 2-class solution was retained because it offered greater parsimony and theoretical coherence. Additional classes represented minor variations of the same dietary patterns, and given the limited number of food items, more complex models would likely capture noise rather than meaningful distinctions. The final model selection prioritised a theoretical interpretability, with class labels defined by the probability of consuming certain food groups.

When applying LTA, three parameters were estimated: the probabilities of belonging to a latent state, the probabilities of item response and the probabilities of transition between latent states. Measurement invariance was tested across the three time points to ensure consistency. In a latent transition model, the inclusion of covariates enables the assessment of their effects on the probability of transitioning from one state to another over time^(18)^. The impact of breast-feeding on dietary transitions was analysed in children with exclusive breast-feeding for 3 to 6 months data, using multinomial logistic regression, and the results are presented as OR with their respective 95 % CI. Mplus version 8.6 was used to implement LCA and LTA^(23)^.

### Multiple imputation method

Due to the profile of the children being followed, analysing data only from children with complete data across all three age groups might result in biased estimates and reduced precision in statistical analyses^(24,25)^. The number and percentage of missing data on food consumption at the considered ages were 51 418 (19 %), 6299 (3 %) and 85 053 (36 %) for early childhood, preschool and school age, respectively. Additionally, 130 685 observations considering exclusive breast-feeding from 3 to 6 months were also missing. Therefore, multiple imputation was chosen as the methodological approach.

Multivariate Imputation by Chained Equations was used to replace missing values based on observed data^(23)^. As the variables were binary (indicating yes *v*. no food consumption on the previous day), we used a logistic regression with binomial distribution family for imputations. The mice package in R was used, with logreg as the method, performing ten imputations per time point^(26)^. Each imputed dataset was analysed using LTA, and results are shown in online supplementary material, Supplemental Table 1. All the variables from food items from all time points and breast-feeding were included in the imputation models. The Rubin’s method was used to obtain the pooled estimates from the LTA model.

A complementary analysis was conducted to compare dietary patterns between the imputed and complete samples. The number of classes was consistent across both samples, with slight differences in class membership in early childhood (see online supplementary material, Supplemental Tables 2, 3, Figure 1). Therefore, the findings are presented using the two-class model based on the imputed data. RStudio version 2023.12.1 and Mplus version 8.6 were used to implement LCA and LTA^(22)^.

## Results

We followed 135 340 children across different life stages. Among them, 50·2 % were female, 40·1 % lived in the Southeast region of Brazil, 46·2 % resided in municipalities with higher levels of deprivation and 26·0 % were exclusively breastfed between 3 and 6 months of age (Table 1).

**Table 1.** Characteristics of the study population, SISVAN, Brazil, 2008–2019 Table 1 long description.

	Total	
Variables	*n*	%
Sex		
Male	67 397	49·8
Female	67 943	50·2
	Mean	sd
Age (months)		
Early childhood	24·8	5·9
Preschool age	48·9	11·1
School age	82·3	7·6
	*n*	%
Macroregion		
North	14 339	10·6
Northeast	37 397	27·6
Southeast	54 303	40·1
South	15 877	11·7
Midwest	10 255	7·6
Missing	3131	2·3
Brazilian Deprivation Index		
1 quintile	7595	5·6
2 quintile	10 149	7·5
3 quintile	18 425	13·5
4 quintile	33 591	24·8
5 quintile	62 586	46·2
Missing	3174	2·3
Food consumption		
Exclusive breast-feeding	35 697	26·4
Fruits		
Early childhood	83 782	61·9
Preschool age	96 614	71·4
School age	103 802	76·7
Vegetables		
Early childhood	82 725	61·1
Preschool age	91 619	67·7
School age	88 507	65·4
Beans		
Early childhood	107 555	79·5
Preschool age	121 547	89·8
School age	117 361	86·7
Sugar-sweetened beverages		
Early childhood	82 184	60·7
Preschool age	96 720	71·5
School age	89 866	66·4
Snacks		
Early childhood	71 374	52·7
Preschool age	76 824	56·8
School age	66 880	49·4
Sweets		
Early childhood	81 870	60·5
Preschool age	90 420	66·8
School age	82 116	60·7

Cross-sectional LCA was fitted separately for each life stage considering the six food groups. Based on model fit indices (see online supplementary material, Supplemental Table 4), three or four classes were identified; however, based on the interpretability criteria and need of study transitions between patterns over time, we chose the model with two latent classes. The first latent class showed a markedly increased probability of consuming ultra-processed food groups, hence named ‘higher ultra-processed food’. The second latent class showed moderate/reduced probabilities of consuming ultra-processed foods, labelled ‘lower ultra-processed food’ (Table 2). Through the likelihood ratio test (*P*-value = 1·0), we did not reject the hypothesis of invariance, supporting the assumption of similarity in the conditional probabilities over time.

**Table 2.** Response probabilities for food groups used to derive the dietary patterns. SISVAN, Brazil, 2008–2019 Table 2 long description.

	Latent classes (dietary patterns)	
	Lower ultra-processed food	Higher ultra-processed food
Fruits	73 %	68 %
Vegetables and/or greens	63 %	67 %
Beans	82 %	88 %
Sweetened beverages	40 %	87 %
Sandwich cookies, sweets or treats	29 %	90 %
Instant noodles, packaged snacks or savoury biscuits	18 %	81 %

**Figure 2.**
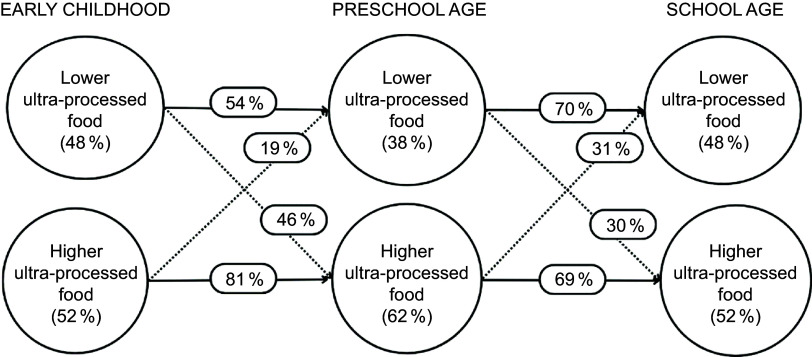
Prevalence of dietary patterns and transition probabilities across early childhood, preschool age and school age, based on the LTA model. SISVAN 2008–2019. Circles represent the latent classes identified at each age group, with the proportion of children in each class. Solid arrows indicate the probability of remaining in the same class over time, whereas dotted arrows indicate the probability of transitioning to another class. LTA, latent transition analysis.

In Figure 2, we present the prevalence for each latent class at the three time points (in parentheses), the probabilities of remaining in the same latent state (solid arrow) and the conditional transition probabilities of belonging to the state at the previous time (dotted arrow). As presented in Figure 2, the prevalence of higher ultra-processed food consumption was elevated across all stages, with a peak in preschool age, reaching 62 %. In early childhood and school age, around half of the children followed a higher ultra-processed food pattern (52 % at both stages). From early childhood to preschool age, 81 % of children in the higher ultra-processed food pattern remained in this group, while 19 % shifted to a lower ultra-processed food pattern. Conversely, 54 % of children showed stability in the lower ultra-processed food pattern over the years, while 46 % transitioned to the higher ultra-processed food pattern from early childhood to preschool age. From preschool to school age, children in both dietary patterns showed greater stability, with 70 % of those in the lower ultra-processed food pattern and 69 % of those in the higher ultra-processed food pattern remaining in their respective groups.

In the analysis focusing on the effect of breast-feeding on the transition between dietary patterns, children who were exclusively breastfed between 3 and 6 months and had a lower ultra-processed food pattern in early childhood had a higher likelihood of remaining in this pattern at preschool age (OR = 1·11; 95 % CI: 1·05, 1·17) and a lower likelihood of transitioning from a reduced to a higher ultra-processed food pattern in this same period (OR = 0·90; 95 % CI: 0·86, 0·95). However, exclusive breast-feeding did not have a significant effect on the stability or transition between dietary patterns over time (Table 3).

**Table 3.** OR and respective 95 % CI for assessing the effect of exclusive breast-feeding on the transition between latent classes over time. SISVAN, Brazil, 2008–2019 Table 3 long description.

		Preschool age				School age			
		Lower ultra-processed food		Higher ultra-processed food		Lower ultra-processed food		Higher ultra-processed food	
		OR	95 % CI	OR	95 % CI	OR	95 % CI	OR	95 % CI
Early childhood	Lower ultra-processed food	**1·11**	**1·05, 1·17**	**0·90**	**0·86, 0·95**	1·03	0·97, 1·11	1·02	0·95, 1·47
	Higher ultra-processed food	1·03	0·97, 1·10	0·97	0·92, 1·03	0·99	0·89, 1·11	1·02	0·97, 1·08

OR: odds ratio; 95%CI: 95% confidence intervals.

## Discussion

In Brazilian children, two main dietary patterns were identified over time according to exposure to ultra-processed food, and our results suggested considerable stability (tracking) from early childhood to school age. Additionally, compared to children who were not exclusively breastfed, children exclusively breastfed for 3–6 months who adopted the lower ultra-processed food pattern in early childhood were less likely to adopt a higher ultra-processed food pattern in the subsequent life stage.

About 52 % of children followed up were classified within the higher ultra-processed food pattern during early childhood and school age. In contrast, those in the lower ultra-processed food pattern represented 48 % in early childhood, decreasing to 38 % at preschool age and then increasing again to 48 % by school age.

These patterns reflect a global trend in children’s food consumption, indicating that dietary habits formed in early childhood often persist into later stages of life^(9,27)^. Similar findings have been reported in countries such as the UK, USA, Canada, Chile, Colombia and Mexico, and other surveys in Brazil showed high consumption of ultra-processed foods^(28–31)^. This highlights the vulnerability of this population group to increasing exposure, partly due to the relatively low price reduction, convenience, high palatability and aggressive marketing of these foods^(32,33)^.

There was a high percentage of children remaining in the same dietary patterns over time, with a retention rate exceeding 54 %. Some studies have investigated the stability of nutrient intake from over time in children^(27,34,35)^. However, few evaluate this stability by food groups or dietary patterns from early childhood to school age^(27,34)^, and much less using the same analytical strategies as our study^(36)^. Studies with distinct methodological strategies, but which also assessed dietary consumption tracking, found stability in dietary intake over time, aligning with the findings of our study^(3,27,34,35)^.

In a Norwegian study, children were assessed at three time points (18 months, 36 months and 7 years) to track the trajectory of low, medium or high consumption of fruits, vegetables and sugary beverages. The results showed that 50 % of children remained in the consumption category observed at age 18 months in later years, at 36 months and 7 years^(34)^. Northstone and Emmett (2013) evaluated dietary patterns at ages 3, 4, 7 and 9 years, categorised as ‘processed’, ‘traditional’ and ‘health-conscious’ and observed that children remained within the same dietary patterns over time^(35)^. These results, suggesting the tracking of dietary patterns across these ages, may be explained by the strong influence of the family on the child’s diet, especially through food purchasing and preparation^(37)^.

Our study found that the percentage of children who adopted the higher ultra-processed food pattern decreased from 62 % at preschool age to 52 % during school age. In addition to children starting to be more involved in their own food choices and showing an increasing desire for independence at this stage, this decrease may be partially attributed to the Brazilian government’s School Feeding Program (PNAE)^(38,39)^. This programme prioritises access to a menu based on healthy, safe, culturally appropriate and sufficient nutrition during the school period, while also promoting nutritional education^(38)^. The consumption of school meals positively impacts the quality of children’s diets, with those who consume more school meals showing a lower intake of ultra-processed foods^(39)^. This contributes to the holistic development of students and the formation of healthy eating habits from childhood. Although there was a decrease in the percentage of children following the higher ultra-processed food pattern in the last two life stages, the proportion of children in the higher ultra-processed food group exceeds half of the studied population in all life stages.

In addition to the PNAE, our findings align with national policies and guidelines aimed at reducing children’s exposure to ultra-processed foods and promoting healthier dietary practices. The Dietary Guidelines for the Brazilian Population (2014) and the Food Guide for Brazilian Children Under Two Years of Age (2019) emphasise the importance of offering unprocessed and minimally processed foods from early childhood, discourage the consumption of ultra-processed foods and recommend the promotion of breast-feeding and healthy complementary feeding^(40,41)^. In this context, our results reinforce the relevance of these actions by demonstrating that exclusive breast-feeding is associated with dietary trajectories characterised by a lower ultra-processed food pattern, while also highlighting persistent challenges, given the high proportion of children who remain in patterns characterised by a higher ultra-processed food pattern.

The high consumption of ultra-processed foods in our study is a cause for concern, as higher ultra-processed food intakes are often associated with poor nutritional quality and may contribute to the risk of overweight and obesity^(31)^. Given the high prevalence of overweight/obesity among children followed up in PHC in Brazil (30 % in boys and 26·6 % in girls aged 5–10 years) in recent years^(4)^, we highlight the importance of investigating dietary patterns, including ultra-processed food consumption, as a factor influencing nutritional and health status.

Additionally, we assessed the effect of exclusive breast-feeding on the transition between dietary patterns. Our findings revealed a protective effect, with exclusively breastfed children more likely to adopt the lower ultra-processed food pattern and less likely to transit to the high ultra-processed pattern by preschool age. This finding suggests that exclusive breast-feeding potentially influences healthier dietary trajectories, not only due to innate taste preferences but also by repeated exposure to specific foods that stimulate their acceptance^(42)^. Studies suggest that exclusive breast-feeding is positively correlated with healthier dietary patterns in later stages of childhood^(43,44)^. Specifically, a longer duration of exclusive breast-feeding is associated with a better dietary pattern in children, characterised by a higher consumption of healthy foods and a lower intake of ultra-processed foods^(42–44)^. Thus, behavioural factors, especially those experienced in the family food environment and during weaning, can influence children’s food intake^(45)^. Children who experience a food environment with positive role models and frequent exposure to healthy foods are more likely to develop healthier eating habits^(37)^. Repeated exposure to healthy foods with variety during the weaning phase increases acceptance of a wider range of foods throughout life^(46)^. Therefore, our findings suggest that promoting healthy eating behaviours at an early age is important to prevent unfavourable eating behaviours later on. Additionally, targeting nutritional interventions for mothers may be an important strategy to improve children’s eating habits.

### Strengths and limitations

Although SISVAN coverage of dietary intake records has increased in recent years, it remains limited, which constrains the representativeness of the data^(47,48)^. Nevertheless, our study analysed more than 135 000 children from all regions of Brazil, which strengthens the robustness of the estimates. In addition, evidence suggests that, despite low coverage, SISVAN data capture patterns that are consistent with those observed in population-based surveys, reinforcing its utility for nutritional surveillance^(49)^.

The availability of repeated observations on food consumption and access to routinely collected data from health service users between 2008 and 2019 provide a unique opportunity to explore dietary patterns among Brazilian children and assess their stability over time. Additionally, we used LTA, a more suitable technique for evaluating the longitudinal transition of these patterns. This approach ensures the assessment of changes over time while addressing measurement invariance, thereby enhancing the robustness and validity of our findings^(19,20)^. It ensures that the observed differences or transitions truly reflect changes in dietary patterns rather than variations in the measurement model itself.

However, there was a significant loss to follow-up, resulting in missing food consumption data, which could introduce information bias. To overcome this limitation, we conducted multiple imputations under the assumption that the data were missing at random^(24)^. This method allowed individuals with data available in only two life stages to be included in the analysis. The imputation process relies on the assumption that the variables included in the model explain the differences between respondents and non-respondents^(24)^. However, it is possible that unmeasured variables influencing these differences were not accounted for, which represents a limitation of our approach.

Although data imputation can introduce some degree of systematic error in parameter estimation, representing a limitation to the approach, studies comparing imputation techniques in longitudinal data have shown that multiple imputation introduces negligible bias when compared to other methods^(25)^. As the differences in consumption probabilities are small between the evaluated life stages, it is believed that the changes are real and not unduly affected by biases. In addition, as observed in supplementary results, the estimates were stable with the increasing number of imputations, which argues in favour of no need of increasing the number of imputations performed (see online supplementary material, Supplemental Table 4).

The breast-feeding variable showed a higher proportion of missing values, which may have introduced some degree of bias. To minimise this limitation, we conducted a sensitivity analysis restricted to the observed breast-feeding data (see online supplementary material, Supplemental Tables 5 and 6). The results remained consistent in direction, with overlapping 95 % CI, indicating stability in the estimates. Nevertheless, these findings should be interpreted as hypothesis-generating and highlight the need for further longitudinal studies to deepen the investigation of this association.

The restricted number of food groups could be considered a limitation for identifying dietary patterns, even though it allows for the routine assessment of children followed in PHC by different health professionals. In addition, our database lacked information on important potential confounding factors, such as socio-economic status and maternal education, which prevented us from performing adjusted analyses and may have limited our ability to fully account for the influence of social and contextual determinants on dietary patterns. Moreover, it has been shown that the internal structure of the dietary intake markers forms adequately reflected its conceptual foundation and that factors related to healthy and unhealthy eating present in the questionnaire maintained stability, acceptable fit indices and metric and scalar invariance, suggesting that the use of this tool is feasible and should be encouraged^(50)^.

We identified two dietary patterns at three developmental stages (1 to 3 years, 3 to < 6 years, 6 to < 11 years): higher ultra-processed food and reduced ultra-processed food. Our results suggest stability (tracking) of dietary patterns over time. Additionally, exclusive breast-feeding was associated with reduced likelihoods of transitioning to a higher ultra-processed food pattern, reinforcing the influence of early nutrition on long-term dietary behaviours. The maintenance of dietary patterns over this age range provides scientific evidence to drive the global discussion on the importance of early dietary education and breast-feeding in promoting healthier and more sustainable dietary habits across the life cycle.

## Supporting information

10.1017/S1368980026102663.sm001 Souza et al. supplementary material Souza et al. supplementary material

## Supplementary material

For supplementary material accompanying this paper visit https://doi.org/10.1017/S1368980026102663

## Table 1. Long description

The table presents characteristics of a study population in Brazil from 2008 to 2019. It has 25 rows and 3 columns. The columns are labeled ‘Variables’, ‘n’, and ‘%’. The rows are grouped under different categories: Sex, Age (months), Macroregion, Brazilian Deprivation Index, and Food consumption. Each row provides specific data points under these categories. For example, under Sex, there are 67397 males (49.8 percent) and 67943 females (50.2 percent). Age is divided into Early childhood, Preschool age, and School age with respective mean ages and percentages. Macroregion includes North, Northeast, Southeast, South, Midwest, and Missing with their respective counts and percentages. Brazilian Deprivation Index is divided into 1 quintile to 5 quintile and Missing with their respective counts and percentages. Food consumption includes categories like Exclusive breast-feeding, Fruits, Vegetables, Beans, Sugar-sweetened beverages, Snacks, and Sweets, each further divided into Early childhood, Preschool age, and School age with their respective counts and percentages.

Navigate back to Table 1..

## Table 2. Long description

A table comparing dietary patterns based on the consumption of ultra-processed foods. The table has two columns and seven rows, including the header. The columns are labeled ‘Lower ultra-processed food’ and ‘Higher ultra-processed food,’ representing different dietary patterns. The rows list different food groups with their respective probabilities of consumption. Row 1: Fruits, 73 percent, 68 percent. Row 2: Vegetables and/or greens, 63 percent, 67 percent. Row 3: Beans, 82 percent, 88 percent. Row 4: Sweetened beverages, 40 percent, 87 percent. Row 5: Sandwich cookies, sweets or treats, 29 percent, 90 percent. Row 6: Instant noodles, packaged snacks or savoury biscuits, 18 percent, 81 percent.

Navigate back to Table 2..

## Table 3. Long description

A table comparing the effect of exclusive breast-feeding on dietary patterns in children. The table has four rows and six columns. The columns are labeled as Preschool age and School age, each with sub-columns for Lower ultra-processed food and Higher ultra-processed food, and each sub-column has OR and 95% CI. The rows are labeled as Early childhood with sub-rows for Lower ultra-processed food and Higher ultra-processed food. Row 1: Lower ultra-processed food, Preschool age, Lower ultra-processed food, OR 1.11, 95% CI 1.05, 1.17, Higher ultra-processed food, OR 0.90, 95% CI 0.86, 0.95. Row 1: Lower ultra-processed food, School age, Lower ultra-processed food, OR 1.03, 95% CI 0.97, 1.11, Higher ultra-processed food, OR 1.02, 95% CI 0.95, 1.47. Row 2: Higher ultra-processed food, Preschool age, Lower ultra-processed food, OR 1.03, 95% CI 0.97, 1.10, Higher ultra-processed food, OR 0.97, 95% CI 0.92, 1.03. Row 2: Higher ultra-processed food, School age, Lower ultra-processed food, OR 0.99, 95% CI 0.89, 1.11, Higher ultra-processed food, OR 1.02, 95% CI 0.97, 1.08.

Navigate back to Table 3..

## References

[1] Ambrosini GL , Emmett PM , Northstone K et al. (2014) Tracking a dietary pattern associated with increased adiposity in childhood and adolescence. Obesity 22, 458–465. 23804590 10.1002/oby.20542 PMC3846445

[2] Mourão E , Gallo CD , Nascimento FA et al. (2020) Tendência temporal da cobertura do Sistema de Vigilância Alimentar e Nutricional entre crianças menores de 5 anos da região Norte do Brasil, 2008–2017 (Temporal trend of Food and Nutrition Surveillance System coverage among children under 5 in the Northern Region of Brazil, 2008–2017). Epidemiol E Serviços Saúde 29, e2019377. doi: 10.5123/S1679-497420200002000266. 32428169

[3] Northstone K , Smith ADAC , Newby PK et al. (2013) Longitudinal comparisons of dietary patterns derived by cluster analysis in 7- to 13-year-old children. Br J Nutr 109, 2050–2058. 23068994 10.1017/S0007114512004072

[4] Santiago-Vieira C , Velasquez-Melendez G , de Cássia Ribeiro-Silva R et al. (2024) Recent changes in growth trajectories: a population-based cohort study of over 5 million Brazilian children born between 2001 and 2014. Lancet Reg Health – Am 32, 100721. 38629028 10.1016/j.lana.2024.100721 PMC11019368

[5] Gasser CE , Kerr JA , Mensah FK et al. (2017) Stability and change in dietary scores and patterns across six waves of the longitudinal study of Australian children. Br J Nutr 117, 1137–1150. 28478764 10.1017/S0007114517000897

[6] Wu CO , Tian X , Tian L et al. (2019) Nonparametric estimation of risk tracking indices for longitudinal studies. Stat Methods Med Res 29, 481–497. 30945590 10.1177/0962280219839427 PMC8674008

[7] van der Nest G , Lima Passos V , Candel MJJM et al. (2020) An overview of mixture modelling for latent evolutions in longitudinal data: modelling approaches, fit statistics and software. Adv Life Course Res 43, 100323. 36726256 10.1016/j.alcr.2019.100323

[8] Bull CJ & Northstone K (2016) Childhood dietary patterns and cardiovascular risk factors in adolescence: results from the Avon Longitudinal Study of Parents and Children (ALSPAC) cohort. Public Health Nutr 19, 3369–3377. 27339189 10.1017/S1368980016001592 PMC5197929

[9] Grulichová M , Zlámal F , Andrýsková L et al. (2021) Dietary pattern longitudinality during 8 years in children: results from the European Longitudinal Study of Pregnancy and Childhood (ELSPAC–CZ). Public Health Nutr 24, 2611–2617. 32580804 10.1017/S1368980020001056 PMC10195423

[10] Oellingrath IM , Svendsen MV & Brantsæter AL (2011) Tracking of eating patterns and overweight – a follow-up study of Norwegian schoolchildren from middle childhood to early adolescence. Nutr J 10, 106. 21978299 10.1186/1475-2891-10-106 PMC3200168

[11] Delormier T , Frohlich KL & Potvin L (2009) Food and eating as social practice – understanding eating patterns as social phenomena and implications for public health. Sociol Health Illn 31, 215–228. 19220802 10.1111/j.1467-9566.2008.01128.x

[12] Rodrigues PRM , Pereira RA , Cunha DB et al. (2012) Factors associated with dietary patterns in adolescents: a school-based study in Cuiabá, Mato Grosso. Rev Bras Epidemiol Braz J Epidemiol 15, 662–674. 10.1590/s1415-790x2012000300019 23090312

[13] Ministério da Saúde, Departamento de Atenção Básica (2015) Marco de Referência da Vigilância Alimentar e Nutricional na Atenção Básica (Framework for Food and Nutrition Surveillance in Primary Health Care). Brasília: Ministério da Saúde

[14] Mrejen M , Cruz MV & Rosa L (2023) O Sistema de Vigilância Alimentar e Nutricional (SISVAN) como ferramenta de monitoramento do estado nutricional de crianças e adolescentes no Brasil (The Food and Nutrition Surveillance System (SISVAN) as a tool to monitor the nutritional status of children and adolescents in Brazil). Cad Saude Publica. Portuguese. 39(1), 39e00169622. doi: 10.1590/0102-311XPT169622 36856228

[15] Ministério da Saúde, Secretaria de Atenção à Saúde, Departamento de Ações Programáticas Estratégicas (2017) Bases para a Discussão da Política Nacional de Promoção, Proteção e Apoio ao Aleitamento Materno (Basis for the Discussion of the National Policy for the Promotion, Protection and Support of Breastfeeding). Brasília: Ministério da Saúde.

[16] Allik M , Leyland A , Ichihara MYT et al. (2020) Creating small-area deprivation indices: a guide for stages and options. J Epidemiol Community Health 74, 20–25. 31630122 10.1136/jech-2019-213255 PMC6929699

[17] Ministério da Saúde, Secretaria de Atenção à Saúd (2015) Orientações para Avaliação de Marcadores de Consumo Alimentar na Atenção Básica (Guidelines for the Assessment of Food Consumption Markers in Primary Health Care). Brasília: Ministério da Saúde.

[18] Mori M , Krumholz HM & Allore HG (2020) Using latent class analysis to identify hidden clinical phenotypes. JAMA 324, 700–701. 32808993 10.1001/jama.2020.2278

[19] Collins LM & Lanza ST (2009) Latent Class and Latent Transition Analysis: With Applications in the Social Behavioral, and Health Sciences. Hoboken, NJ: John Wiley & Sons. 285.

[20] Naldi L & Cazzaniga S (2020) Research techniques made simple: latent class analysis. J Invest Dermatol 140, 1676–1680.e1. 32800180 10.1016/j.jid.2020.05.079

[21] Collins LM & Wugalter SE (1992) Latent class models for stage-sequential dynamic latent variables. Multivar Behav Res 27, 131–157.

[22] Muthén BO & Muthén LK (2000) Integrating person-centered and variable-centered analyses: growth mixture modeling with latent trajectory classes. Alcohol Clin Exp Res 24, 882–891. 10888079

[23] van Buuren S & Groothuis-Oudshoorn K (2011) mice: multivariate imputation by chained equations in R. J Stat Softw 45, 1–67.

[24] Biering K , Hjollund NH & Frydenberg M (2015) Using multiple imputation to deal with missing data and attrition in longitudinal studies with repeated measures of patient-reported outcomes. Clin Epidemiol 7, 91–106. 25653557 10.2147/CLEP.S72247 PMC4303367

[25] Gad AM & Abdelkhalek RHM (2017) Imputation methods for longitudinal data: a comparative study. Int J Stat Distrib Appl 3, 72.

[26] Carpenter JR & Kenward MG (2012) Multiple Imputation and its Application, pp. 1–345. Chichester, West Sussex: John Wiley and Sons Ltd.

[27] Luque V , Escribano J , Closa-Monasterolo R et al. (2018) Unhealthy dietary patterns established in infancy track to mid-childhood: the EU Childhood Obesity Project. J Nutr 148, 752–759. 29982656 10.1093/jn/nxy025

[28] Lauria F , Russo MD , Formisano A et al. (2021) Ultra-processed foods consumption and diet quality of European children, adolescents and adults: results from the I.Family study. Nutr Metab Cardiovasc Dis 31, 3031–3043. 34518085 10.1016/j.numecd.2021.07.019

[29] Lopes WC , Pinho LD , Caldeira AP et al. (2020) Consumption of ultra-processed foods by children under 24 months of age and associated factors. Rev Paul Pediatr 38, e2018277. 32074226 10.1590/1984-0462/2020/38/2018277 PMC7025446

[30] Neri D , Martinez-Steele E , Monteiro CA et al. (2019) Consumption of ultra-processed foods and its association with added sugar content in the diets of US children, NHANES 2009–2014. Pediatr Obes 14, e12563. 31364315 10.1111/ijpo.12563

[31] Oliveira PG , Sousa JM , Assunção DG et al. (2022) Impacts of consumption of ultra-processed foods on the maternal-child health: a systematic review. Front Nutr 9, 821657. 35634416 10.3389/fnut.2022.821657 PMC9136982

[32] Monteiro CA , Cannon G , Levy RB et al. (2019) Ultra-processed foods: what they are and how to identify them. Public Health Nutr 22, 936–941. 30744710 10.1017/S1368980018003762 PMC10260459

[33] Passos CM , Maia e.g. Levy RB et al. (2020) Association between the price of ultra-processed foods and obesity in Brazil. Nutr Metab Cardiovasc Dis 30, 589–598. 32139251 10.1016/j.numecd.2019.12.011

[34] Bjelland M , Brantsæter AL , Haugen M et al. (2013) Changes and tracking of fruit, vegetables and sugar-sweetened beverages intake from 18 months to 7 years in the Norwegian mother and child cohort study. BMC Public Health 13, 1–11. 24103398 10.1186/1471-2458-13-793 PMC3765981

[35] Northstone K & Emmett PM (2008) A comparison of methods to assess changes in dietary patterns from pregnancy to 4 years post-partum obtained using principal components analysis. Br J Nutr 99, 1099. 17916275 10.1017/S0007114507842802 PMC2493053

[36] Pitt E , Cameron CM , Thornton L et al. (2018) Dietary patterns of Australian children at three and five years of age and their changes over time: a latent class and latent transition analysis. Appetite 129, 207–216. 30012352 10.1016/j.appet.2018.07.008

[37] Scaglioni S , Cosmi VD , Ciappolino V et al. (2018) Factors influencing children’s eating behaviours. Nutrients 10, 706. 29857549 10.3390/nu10060706 PMC6024598

[38] Kroth DC , Geremia DS & Mussio BR (2020) National school feeding program: a healthy public policy. Cienc Saude Coletiva 25, 4065–4076. 10.1590/1413-812320202510.31762018 32997036

[39] Boklis-Berer M , Rauber F , Azeredo CM et al. (2021) The adherence to school meals is associated with a lower occurrence of obesity among Brazilian adolescents. Prev Med 150, 106709. 34181943 10.1016/j.ypmed.2021.106709

[40] Ministério da Saúde (2019) Guia Alimentar para Crianças Brasileiras Menores de 2 Anos (Dietary Guidelines for Brazilian Children Under 2 Years of Age). Brasília: Ministério da Saúde

[41] Ministério da Saúde, Secretaria de Atenção à Saúde, Departamento de Atenção Básica (2014) Guia Alimentar para a População Brasileira (Dietary Guidelines for the Brazilian Population), 2nd ed. Brasília: Ministério da Saúde.

[42] Carvalho CA , Viola PC , Magalhães EI et al. (2024) Association between breast feeding and food consumption according to the degree of processing in Brazil: a cohort study. BMJ Open 14, e083871. 10.1136/bmjopen-2024-083871 PMC10989181 38569686

[43] Kheir F , Feeley N , Maximova K et al. (2021) Breastfeeding duration in infancy and dietary intake in childhood and adolescence. Appetite 158, 104999. 33058954 10.1016/j.appet.2020.104999

[44] Olid AO , Moreno-Galarraga L , Moreno-Villares JM et al. (2023) Breastfeeding is associated with higher adherence to the Mediterranean diet in a Spanish population of preschoolers: the SENDO project. Nutrition 15, 1278. 10.3390/nu15051278 PMC10005753 36904277

[45] Blissett J & Fogel A (2013) Intrinsic and extrinsic influences on children’s acceptance of new foods. Physiol Behav 121, 89–95. 23458629 10.1016/j.physbeh.2013.02.013

[46] Pearce J & Langley-Evans SC (2013) The types of food introduced during complementary feeding and risk of childhood obesity: a systematic review. Int J Obes 37, 477–485. 10.1038/ijo.2013.8 23399778

[47] Nascimento FA , Silva SA & Jaime PC (2019) Cobertura da avaliação do consumo alimentar no Sistema de Vigilância Alimentar e Nutricional Brasileiro: 2008 a 2013 (Coverage of food intake assessment in the Brazilian Food and Nutrition Surveillance System: 2008 to 2013). Rev Bras Epidemiol Braz J Epidemiol 22, e190028. doi: 10.1590/1980-549720190028. 30942334

[48] Ricci JMS , Romito ALZ , Silva SA et al. (2023) Food intake markers in Sisvan: temporal trends in coverage and integration with e-SUS APS, Brazil 2015–2019. Cienc Saude Coletiva 28, 921–934. 10.1590/1413-81232023283.10552022 36888874

[49] Carvalho RB , Louzada ML , Rauber F et al. (2023) Characteristics associated with dietary patterns in Brazilian children under two years of age. Rev Saude Publica 56, 118. 36629709 10.11606/s1518-8787.2022056003757 PMC9749735

[50] Lourenço BH , Guedes BD & Santos TSS (2023) Marcadores do consumo alimentar do Sisvan: estrutura e invariância de mensuração no Brasil (Sisvan food intake markers: structure and measurement invariance in Brazil). Rev Saude Publica 57, 52. doi: 10.11606/S1518-8787.2023057004896. 37585951 PMC10421608

