# Supplementary material for: Tracking of dietary patterns from early childhood to school age in the Brazilian Food and Nutritional Surveillance System, 2008–2019
Source: Public Health Nutr. 2026 Jul 6;29(1):e162. doi: 10.1017/S1368980026102663 (PMC13440020; doi:10.1017/S1368980026102663)
Supplement: Supplementary file 1 [file S1368980026102663sup001.docx]

**SUPPLEMENTARY TABLES**

**Supplementary Table 1 - Item response of conditional probabilities for dietary intake for imputed data. SISVAN, Brazil, 2008 to 2019.**

|  | Mean of imputed data | | Imput 1 | | Imput 2 | | Imput 3 | | Imput 4 | | Imput 5 | | Imput 6 | | Imput 7 | | Imput 8 | | Imput 9 | | Imput 10 | |
| --- | --- | --- | --- | --- | --- | --- | --- | --- | --- | --- | --- | --- | --- | --- | --- | --- | --- | --- | --- | --- | --- | --- |
|  | Lower ultra processed food | Higher ultra processed food | Lower ultra processed food | Higher ultra processed food | Lower ultra processed food | Higher ultra processed food | Lower ultra processed food | Higher ultra processed food | Lower ultra processed food | Higher ultra processed food | Lower ultra processed food | Higher ultra processed food | Lower ultra processed food | Higher ultra processed food | Lower ultra processed food | Higher ultra processed food | Lower ultra processed food | Higher ultra processed food | Lower ultra processed food | Higher ultra processed food | Lower ultra processed food | Higher ultra processed food |
| Fruits | 73% | 68% | 73% | 68% | 73% | 67% | 73% | 68% | 73% | 68% | 73% | 67% | 73% | 68% | 73% | 68% | 73% | 68% | 72% | 68% | 73% | 68% |
| Vegetables and/or greens | 63% | 67% | 62% | 69% | 63% | 67% | 63% | 67% | 63% | 67% | 63% | 67% | 62% | 67% | 62% | 67% | 63% | 67% | 63% | 67% | 62% | 67% |
| Beans | 82% | 88% | 82% | 88% | 83% | 88% | 82% | 88% | 82% | 88% | 82% | 88% | 82% | 88% | 82% | 88% | 82% | 88% | 82% | 88% | 82% | 88% |
| Sweetened beverages | 40% | 87% | 40% | 87% | 41% | 87% | 40% | 87% | 41% | 87% | 41% | 87% | 40% | 87% | 41% | 87% | 40% | 87% | 41% | 87% | 41% | 87% |
| Filled biscuits, sweets or treats | 29% | 90% | 29% | 90% | 29% | 90% | 29% | 90% | 29% | 90% | 29% | 90% | 29% | 90% | 29% | 90% | 29% | 90% | 29% | 90% | 29% | 90% |
| Instant noodles, packaged snacks or savory biscuits | 18% | 81% | 18% | 81% | 18% | 81% | 18% | 81% | 18% | 81% | 18% | 81% | 18% | 81% | 18% | 82% | 18% | 81% | 18% | 81% | 18% | 81% |
| OR of exclusive breastfeeding transition between Early childhood and Preschool age | | | | | | | | | | | | | | | | | | | | | | |
| Lower ultra processed food to Higher ultra processed food | 0.90 | | 0.90 | | 0.87 | | 0.78 | | 0.85 | | 0.98 | | 0.96 | | 1.00 | | 0.90 | | 0.92 | | 0.89 | |
| Lower ultra processed food to lower ultra processed food | 1.11 | | 1.11 | | 1.15 | | 1.28 | | 1.17 | | 1.01 | | 1.05 | | 1.00 | | 1.11 | | 1.09 | | 1.13 | |
| Higher ultra processed food to Lower ultra processed food | 1.03 | | 1.02 | | 1.17 | | 1.06 | | 1.07 | | 0.97 | | 0.94 | | 0.97 | | 1.04 | | 1.06 | | 1.04 | |
| Higher ultra processed food to Higher ultra processed food | 0.97 | | 0.99 | | 0.86 | | 0.95 | | 0.94 | | 1.03 | | 1.07 | | 1.04 | | 0.96 | | 0.94 | | 0.96 | |
| OR of exclusive breastfeeding transition between Preschool age and School age | | | | | | | | | | | | | | | | | | | | | | |
| Lower ultra processed food to Higher ultra processed food | 1.02 | | 1.21 | | 0.80 | | 1.14 | | 0.81 | | 1.42 | | 0.91 | | 0.82 | | 1.11 | | 0.94 | | 1.01 | |
| Lower ultra processed food to lower ultra processed food | 1.03 | | 1.11 | | 1.25 | | 0.87 | | 1.24 | | 0.70 | | 1.10 | | 1.21 | | 0.90 | | 0.95 | | 0.99 | |
| Higher ultra processed food to Lower ultra processed food | 0.99 | | 0.87 | | 1.08 | | 0.96 | | 1.17 | | 0.71 | | 1.13 | | 1.11 | | 0.86 | | 1.06 | | 1.00 | |
| Higher ultra processed food to Higher ultra processed food | 1.02 | | 0.99 | | 0.93 | | 1.04 | | 0.86 | | 1.41 | | 0.88 | | 0.90 | | 1.16 | | 1.06 | | 1.00 | |

| Supplementary Table 2 - Goodness-of-fit statistics for LCA models with different numbers of classes for complete data analysis. | | | |
| --- | --- | --- | --- |
| NUMBER OF CLASSES | 2 | 3 | 4 |
| ENTROPY | 0.63 | **0.70** | 0.68 |
| AIC | 75536 | 72455 | **72181** |
| BIC | 75641 | 72989 | **72498** |

| Supplementary Table 3 - Item response of conditional probabilities for dietary intake for complete data analysis. SISVAN, Brazil, 2008 to 2019. | | |
| --- | --- | --- |
|  | Latent Classes (dietary patterns) | |
| Dietary intake items | **Lower ultra processed food** | **Higher ultra processed food** |
| Fresh fruits | 70% | 52% |
| Vegetables and/or greens | 61% | 67% |
| Beans | 80% | 91% |
| sweetened beverages | 50% | 92% |
| filled biscuits, sweets or treats | 42% | 95% |
| instant noodles, packaged snacks or savory biscuits | 28% | 92% |

Supplementary figure 1. Prevalence of latent states and transition probabilities in the LTA model in complete data analysis. SISVAN 2008 – 2019

Circles represent the latent classes identified at each age group, with the proportion of children in each class. Solid arrows indicate the probability of remaining in the same class over time, whereas dotted arrows indicate the probability of transitioning to another class.

| Supplementary Table 4 - Goodness-of-fit statistics for LCA models with different numbers of classes for imputed data | | | |
| --- | --- | --- | --- |
| NUMBER OF CLASSES | 2 | 3 | 4 |
| ENTROPY | 0.63 | **0.65** | 0.62 |
| AIC | 2823640 | 2753029 | **2735033** |
| BIC | 2823856 | 2753873 | **2736476** |

| Supplementary Table 5 – Item response of conditional probabilities for dietary intake among children with observed breastfeeding data. SISVAN, Brazil, 2008 to 2019. | | |
| --- | --- | --- |
|  | Latent Classes (dietary patterns) | |
| Dietary intake items | Lower ultra processed food | Higher ultra processed food |
| Fresh fruits | 77.5% | 74.4% |
| Vegetables and/or greens | 66.8% | 67.4% |
| Beans | 84.5% | 86.7% |
| sweetened beverages | 32.7% | 83.4% |
| filled biscuits, sweets or treats | 19.9% | 84.6% |
| instant noodles, packaged snacks or savory biscuits | 11.0% | 70.6% |

| **Supplementary Table 6. Odds ratios (OR) and 95% confidence intervals (95% CI) for the effect of exclusive breastfeeding on transitions between latent dietary classes over time among children with observed breastfeeding data. SISVAN, Brazil, 2008–2019.** | | | | | |
| --- | --- | --- | --- | --- | --- |
|  |  | Preschool age | | School age | |
|  |  | Lower ultra processed food  OR (95% CI) | Higher ultra processed food OR (95% CI) | Lower ultra processed food OR (95% CI) | Higher ultra processed food OR (95% CI) |
|  |  |  |  |  |  |
| Early childhood | Lower ultra processed food | **1.34**  **(1.07 – 1.68)** | **0.75**  **(0.60 – 0.93)** | 0.99  (0.72 – 1.36) | 1.01  (0.73 – 1.38) |
|  | Higher ultra processed food | 0.89  (0.62 – 1.28) | 1.12  (0.78 – 1.61) | 0.88  (0.65 – 1.17) | 1.14  (0.85 – 1.53) |
